# Risk of Cardiovascular Events in Metabolically Healthy Overweight or Obese Adults: Role of LDL-Cholesterol in the Stratification of Risk

**DOI:** 10.3390/diagnostics14131314

**Published:** 2024-06-21

**Authors:** Paolo Palatini, Agostino Virdis, Stefano Masi, Alessandro Mengozzi, Edoardo Casiglia, Valerie Tikhonoff, Arrigo F. G. Cicero, Andrea Ungar, Gianfranco Parati, Giulia Rivasi, Massimo Salvetti, Carlo Maria Barbagallo, Michele Bombelli, Raffaella Dell’Oro, Berardino Bruno, Luciano Lippa, Lanfranco D’Elia, Maria Masulli, Paolo Verdecchia, Gianpaolo Reboldi, Fabio Angeli, Rosario Cianci, Francesca Mallamaci, Massimo Cirillo, Marcello Rattazzi, Pietro Cirillo, Loreto Gesualdo, Elisa Russo, Alberto Mazza, Cristina Giannattasio, Alessandro Maloberti, Massimo Volpe, Giuliano Tocci, Guido Iaccarino, Pietro Nazzaro, Ferruccio Galletti, Claudio Ferri, Giovambattista Desideri, Francesca Viazzi, Roberto Pontremoli, Maria Lorenza Muiesan, Guido Grassi, Claudio Borghi

**Affiliations:** 1 Department of Medicine, Studium Patavinum, University of Padova, 35128 Padua, Italy; 2 Department of Clinical and Experimental Medicine, University of Pisa, 56126 Pisa, Italy; 3 Department of Medicine, University of Padova, 35128 Padua, Italy; 4 Alma Mater Studiorum, University of Bologna, 40126 Bologna, Italy; 5 Department of Geriatric and Intensive Care Medicine, Careggi Hospital, University of Florence, 50121 Florence, Italy; 6 Istituto Auxologico Italiano, S. Luca Hospital, University of Milan-Bicocca, 20126 Milan, Italy; gianfranco.parati@unimib.it; 7 Department of Medicine and Surgery, University of Milano-Bicocca, Piazza dell’Ateneo Nuovo, 20126 Milan, Italy; 8 Department of Clinical and Experimental Sciences, University of Brescia, 25123 Brescia, Italy; 9 Biomedical Department of Internal Medicine and Specialistics, University of Palermo, 90100 Palermo, Italy; 10 Department of Medicine and Surgery, University of Milano-Bicocca, 20900 Monza, Italyraffaella.delloro@unimib.it (R.D.); guido.grassi@unimib.it (G.G.); 11 Department of Life, Health and Environmental Sciences, University of L’Aquila, 67100 L’Aquila, Italy; 12 Italian Society of General Medicine (SIMG), 67051 Avezzano, Italy; 13 Department of Clinical Medicine and Surgery, Medical School, “Federico II” University of Naples, 80133 Naples, Italy; 14 Hospital S. Maria della Misericordia, 06100 Perugia, Italy; 15 Department of Medical and Surgical Science, University of Perugia, 06100 Perugia, Italy; paolo.reboldi@unipg.it; 16 Department of Medicine and Surgery, University of Insubria, 21100 Varese, Italy; 17 Department of Medicine and Cardiopulmonary Rehabilitation, Maugeri Care and Research Institutes, IRCCS, 21100 Varese, Italy; 18 Department of Translational and Precision Medicine, Sapienza University of Rome, 00185 Rome, Italy; 19 CNR-IFC, Clinical Epidemiology of Renal Diseases and Hypertension, Reggio Cal Unit, 89124 Reggio Calabria, Italy; 20 Department of Public Health, “Federico II” University of Naples, 80133 Naples, Italy; mcirillo@unisa.it; 21 Medicina Interna 1°, Ca’ Foncello University Hospital, 31100 Treviso, Italy; 22 Nephrology, Dialysis and Transplantation Unit, Department of Emergency and Organ Transplantation, “Aldo Moro” University of Bari, 70122 Bari, Italy; cirillo.pietro@yahoo.it (P.C.);; 23 Department of Internal Medicine, University of Genoa, Policlinico San Martino, 16132 Genova, Italyfrancesca.viazzi@unige.it (F.V.);; 24 Department of Internal Medicine, Hypertension Unit, General Hospital, 45100 Rovigo, Italy; alberto.mazza@aulss5.veneto.it; 25 Cardiology IV, ‘A. De Gasperis’ Department, Niguarda Ca’ Granda Hospital, 20162 Milano, Italy; 26 Department of Clinical and Molecular Medicine, University of Rome Sapienza, 00161 Rome, Italy; 27 IRCCS San Raffaele, 00161 Rome, Italy; 28 Department of Clinical and Molecular Medicine, Faculty of Medicine and Psychology, University of Rome Sapienza, Sant’Andrea Hospital, 00185 Rome, Italy; giuliano.tocci@uniroma1.it; 29 Department of Advanced Biomedical Sciences, “Federico II” University of Naples, 80133 Naples, Italy; 30 Department of Medical Basic Sciences, Neurosciences and Sense Organs, Medical School, University of Bari, 70122 Bari, Italy; pietronazzaro@gmail.com

**Keywords:** obesity, metabolically healthy, LDL-cholesterol, cardiovascular, events

## Abstract

The objective of this study was to investigate the longitudinal association of metabolically healthy overweight/obese adults with major adverse cardiovascular events (MACE) and the effect of LDL-cholesterol levels on this association. This study was conducted with 15,904 participants from the URRAH study grouped according to BMI and metabolic status. Healthy metabolic status was identified with and without including LDL-cholesterol. The risk of MACE during 11.8 years of follow-up was evaluated with multivariable Cox regressions. Among the participants aged <70 years, high BMI was associated with an increased risk of MACE, whereas among the older subjects it was associated with lower risk. Compared to the group with normal weight/healthy metabolic status, the metabolically healthy participants aged <70 years who were overweight/obese had an increased risk of MACE with an adjusted hazard ratio of 3.81 (95% CI, 1.34–10.85, *p* = 0.012). However, when LDL-cholesterol < 130 mg/dL was included in the definition of healthy metabolic status, no increase in risk was found in the overweight/obese adults compared to the normal weight individuals (hazard ratio 0.70 (0.07–6.71, *p* = 0.75). The present data show that the risk of MACE is increased in metabolically healthy overweight/obese individuals identified according to standard criteria. However, when LDL-cholesterol is included in the definition, metabolically healthy individuals who are overweight/obese have no increase in risk.

## 1. Introduction

A large number of studies have shown that a subgroup of individuals who are overweight or obese (OwOb) is characterized by the absence of metabolic abnormalities and may be protected from obesity-related cardiovascular (CV) diseases [1,2,3,4,5,6]. Although this subset of individuals is at lower CV risk than individuals who are metabolically unhealthy while OwOb, the actual impact of metabolically healthy obesity (MHO) on CV disease is still controversial. According to many investigators, a metabolically healthy profile does not completely protect people who are OwOb from CV events [1,2,5,6]. This controversy is partly due to the inconsistent definition of metabolic health and the frequent progression of MHO towards unhealthy metabolic status over time. Although in all investigations individuals with MHO had a better metabolic profile than those with metabolically unhealthy obesity, in some studies individuals with one or two metabolic abnormalities were included among the “healthy” participants [1,2,3,4,5,6].

In recent years, stricter criteria were used to identify people with healthy metabolic status. In agreement with Lavie et al. [3], in the present study, healthy metabolic status was defined as a normal BP and the absence of any abnormal metabolic parameter (fasting glucose < 100 mg/dL, triglyceride < 150 mg/dL, and high-density lipoprotein cholesterol (HDL-cholesterol) ≥ 40 mg/dL in men and ≥50 mg/dL in women).

Several studies have shown that healthy metabolic status is not a stable condition and that many subjects with MHO develop abnormal metabolic parameters transitioning to a metabolically unhealthy obese status [7,8]. According to recent results obtained within the frame of the HARVEST study, only one third of participants with metabolically healthy OwOb status remained metabolically healthy after 7.5 years of observation [9]. These findings suggest that many individuals with MHO are at an intermediate stage of progression towards an unhealthy metabolic status. It would thus be crucial to identify factors that better identify a true healthy metabolic state in OwOb individuals and can predict the transition to an unhealthy state over time. Indeed, the characteristics of MHO remain to be established. Increased inflammation has been observed in patients with an unhealthy metabolic state and low levels of inflammatory markers may contribute to identifying the MHO phenotype [10,11,12]. LDL-C may not only have a direct effect on the arterial wall, promoting atherosclerotic lesions, but also acts as a moderator between other risk factors and adverse cardiovascular outcomes. In a study by Zhang M. et al. in which LDL-C was included in the MHO definition, a metabolically healthy status was associated with a low inflammatory state [13]. In a study performed in Japanese people, Oda et al. showed that LDL-cholesterol (LDL-C) was related to metabolic syndrome and can serve as a predictor for its development [14]. Although LDL-C is a major risk factor for atherosclerosis and CV events, it was rarely considered among the factors influencing the association of unhealthy obesity with adverse outcomes in MHO studies.

Another factor to consider within obesity research is the role of age. In the elderly, there are situations where being OwOb appear to be beneficial. This so-called obesity paradox appears to confer a survival advantage in older patients, especially in the presence of chronic diseases [15,16]. Based on this premise, it is logical to expect that the relationship of BMI and/or metabolic status with adverse outcomes will be neutral or negative in older subjects.

The aim of the present study was to investigate the risk of major adverse CV events (MACE) associated with OwOb status in the participants of the Uric Acid Right for Heart Health (URRAH) study [17,18], stratified according to OwOb and metabolic status. The URRAH participants are patients with hypertension or high cardiovascular risk currently referred to hospital centres [17]. This subset of individuals has a higher likelihood of having metabolic abnormalities compared to people from general populations currently included in MHO studies [1,2] and may have a different relationship of metabolic status with CV outcomes. Thus, the aim of the present study was to evaluate the prognostic significance of MHO within a population of hypertensive and high-risk patients referred to tertiary centres. One main purpose was to examine the role of LDL-C in the definition of MHO and its impact on the MHO–MACE association in this sample. To this purpose, LDL-C was used either as a predictor of MACE in the survival model or to refine the definition of MHO in order to identify the participants with true healthy OwOb status. Due to the different relationship of OwOb status with CV outcomes in adults and older subjects, the prognostic role of OwOb/metabolic status was tested in the participants grouped according to age.

## 2. Material and Methods

### 2.1. Participants

The URRAH project is a multicentre retrospective, observational study promoted by the Italian Society of Hypertension [17,18]. Aim of the URRAH project is to study the relationship of uric acid and metabolic variables with CV disease within a large sample of subjects aged 18–95 years with hypertension or high cardiovascular risk referred to tertiary centres and collected on a regional basis. The database was constructed by merging data from Caucasian subjects recruited in prospective observational cohort studies as well as patients attending hypertension clinics up to 31 July 2017. Centres of Hypertension distributed in almost all the Italian regions participated in the study. Patients with previous CV events were excluded. All procedures were in accordance with the ethical standards of the institutional and/or national research committee and with the 1964 Helsinki Declaration and its later amendments or comparable ethical standards. The study protocol was approved by the local ethics committees in all participating centres, and written informed consent was obtained from all of the participants.

For the present study, we selected 15,904 participants (8284 women) in whom metabolic variables, LDL-C, uric acid, creatinine, office blood pressure (BP), and information on cardiovascular risk factors and MACE during the follow-up were available.

### 2.2. Procedures

All participants underwent physical examination, anthropometry, and blood chemistry after an overnight fast. Biochemical tests were measured using standard methods. Diabetes mellitus was defined by treatment with antidiabetic drugs, fasting plasma glucose ≥ 6.99 mmol/L (126 mg/dL), or haemoglobin A1c ≥ 48 mmol/mol (≥6.5%) [19]. LDL-C was calculated as follows: total cholesterol minus HDL-C levels, minus triglyceride level divided by 5, if the triglyceride level was less than 400 mg/dL. Participants were classified into three BP categories according to the 2023 ESH Guidelines (normotensives, stage 1 hypertensives, and stage 2 hypertensives) [20]. Antihypertensive treatment was taken by 31.5% of the participants and lipid lowering drugs by 8.0%. MACE included fatal and non-fatal events due to acute myocardial infarction, heart failure or stroke, and sudden cardiac death. Information about MACE was obtained from hospital records or death certificates. Survival time for the participants was defined as the period from the date of the first visit of the participant to the date of first MACE. Other details of the URRAH project and the procedures used to measure covariates have been previously published [17,18].

### 2.3. Classification of Participants by BMI and Metabolic Status

Normal body weight was defined as a BMI < 25 kg/m^2^, overweight as a BMI from 25 to 29.9 kg/m^2^, and obesity as a BMI ≥ 30 kg/m^2^. In the survival analyses, overweight and obese were first considered as separate categories and then lumped together into a single OwOb group. High waist circumference was defined based on current diagnostic criteria for metabolic syndrome (≥102 cm for men and ≥88 cm for women) [20]. Healthy metabolic status was identified using two definitions: (1) standard criteria (triglyceride levels < 150 mg/dL, high-density lipoprotein cholesterol (HDL-cholesterol) ≥ 40 mg/dL in men and ≥50 mg/dL in women, fasting glucose < 100 mg/dL, and office systolic BP < 130/85 mmHg); (2) the above criteria plus a level of LDL-C < 130 mg/dL [21]. Only subjects without diabetes were included in the metabolically healthy subgroups.

### 2.4. Statistics

Quantitative variables were reported as mean and SD and differences in the distribution across groups were tested by ANCOVA test adjusting for age and sex. Differences in mean values were tested with an unpaired Student *t* test. The Chi-squared test was used to evaluate differences between categorical variables. The association between BMI category and time to MACE was analyzed in the participants stratified by age (<70 or ≥70 years) using the Cox proportional hazards regression model and adjusting for sex (model 1) and then by age, sex, smoking and former smoking, metabolic status, LDL-C, uric acid, and creatinine (model 2). In the participants aged <70 years, the predictive value of the BMI group/metabolic status combination was tested in models adjusted for confounders and other risk factors (age, sex, smoking and former smoking, LDL-C, uric acid, and creatinine), which was the main multivariable model. Further adjustments were made for antihypertensive treatment and BP category to see if these were confounders for the association between BMI/metabolic status group and MACE. Creatinine and uric acid were included in the survival models because, in the URRAH study, they proved to be strong predictors of adverse outcomes [17,18]. For models including waist circumference, physical activity habits, and alcohol consumption, missing data were handled by means of deletion methods; hence, models were based on fewer observations (see Section 3). In all Cox regressions, the risk of MACE was tested either including or excluding the patients with diabetes in the metabolically unhealthy subgroups. We used a Wald test for the regression coefficients to test the null hypothesis that risk factors had no effect. The hazard ratios (HRs) from the multivariable analyses and their corresponding two-sided 95% confidence intervals (CI) were derived from the regression coefficients in the Cox models. To compare non-nested models, with or without LDL-C, we used the Akaike Information Criteria (AIC) and the Schwarz’s Bayesian Information Criterion (BIC) [22]. For both AIC and BIC, the model providing smallest values is considered to have the best fit. A measure that can be used to compare survival models is the delta AIC. As a rule of thumb for interpretation, a delta AIC < 2 suggests substantial evidence for the model, and values between 3 and 7 indicate that the model has considerably less support [22]. In an additional Cox model, the metabolically healthy participants were identified using the standard criteria plus a level of LDL-C < 130 mg/dL, and the unhealthy participants were identified by including a level of LDL-C ≥ 130 mg/dL. In this model, LDL-C was no longer included as a covariate. To evaluate the potential modifying effects of hypertension on the relationship between LDL-C exposure and CV events, we created two interaction terms between BP status (hypertension/normotension) and LDL-C category (high/low) and between BP level (high/low) and LDL-C category, which were tested separately within the model described above.

Analyses were performed using a significance level of α = 0.05 (2-sided). Statistical analysis was carried out using SYSTAT version 12 (SPSS Inc., Chicago, IL, USA) and Medcalc version 20.014 (Ostend, Belgium) packages.

## 3. Results

The characteristics of the participants stratified by age category are reported in Table S1. Compared with the subjects <70 years of age, the older participants were more frequently female, hypertensive, and diabetic. They had higher office BP, waist circumference, glucose, uric acid, and HDL-cholesterol and lower LDL-C. BMI was slightly higher among the older subjects and had a similar distribution in the two groups, with positive skewness and negative Kurtosis (Figure S1). Diabetes mellitus was present in 6.0% of the subjects younger than 70 years and in 16.6% of those ≥ 70 years. During a median 11.4-year (interquartile range, 5.3–13.3 years) follow-up, there were 1752 cases (11.0%) of MACE in the whole population: 6.1% among the participants <70 years and 24.2% among those ≥70 years.

**Figure 1:**
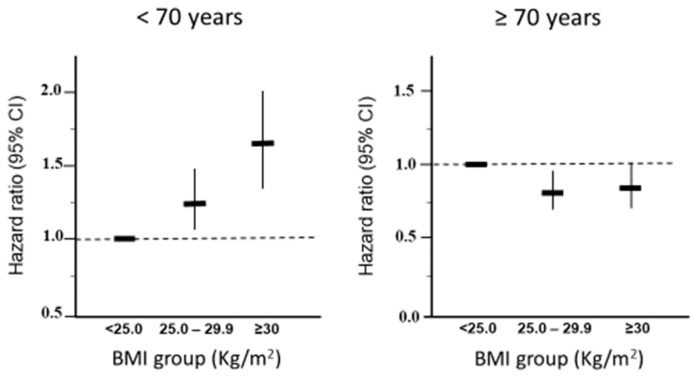
Sex-adjusted hazard ratios and 95% confidence intervals for risk of major adverse cardiovascular events according to body mass index (BMI) category in 15,904 URRAH participants stratified by age.

In a sex-adjusted Cox regression, the association of BMI with the risk of MACE had opposite associations in the two age groups (Figure 1). Among the participants <70 years, the risk of MACE was increased in both groups who were OwOb, whereas higher BMI was associated with lower risk among the older subjects. Similar results were obtained in a multivariable model adjusted for age, sex, smoking, metabolic status, total cholesterol, serum creatinine, and uric acid (Table S2).

A different age-related association with CV risk was found for metabolic status. In the <70-years-old group, the sex-adjusted risk of MACE was increased in the participants with an unhealthy metabolic status (HR, 6.48; 95% CI, 4.01–10.49, *p* < 0.001), even when data were adjusted for BMI (HR, 5.45; 95% CI, 3.36–8.86, *p* < 0.001). In contrast, no association was found in the participants ≥70 years for both sex-adjusted data (HR, 1.16; 95% CI, 0.76–1.75, *p* = 0.49) and data adjusted for age, sex, smoking, metabolic status, total cholesterol, serum creatinine, and uric acid (HR, 1.23; 95% CI, 0.82–1.87, *p* = 0.32). Thus, the CV risk of BMI according to metabolic status was tested only in the participants younger than 70 years.

### 3.1. CV Risk Associated with BMI and Metabolic Status Combined in the Participants <70 Years

Of the 11,585 participants younger than 70 years, 39.6% had a normal weight, 42.0% were overweight, and 18.4% had obesity. A healthy metabolic status was present in 12.4%. BMI distribution according to metabolic status is presented in Figure S2. In both subgroups, the distribution was skewed to the right. BMI was correlated with glucose (R = 0.24), triglycerides (R = 0.23), and HDL-cholesterol (R = −0.23), all *p* < 0.0001. A weaker correlation was found with LDL-C (R = 0.09, *p* < 0.001). A strong correlation was found with waist circumference (N = 10,112, R = 0.75, *p* < 0.0001). Among the participants with an unhealthy metabolic status, high BP was present in 67.3% of the subjects, high glucose in 23.5%, high triglycerides in 21.7%, and low HDL-cholesterol in 28.6%.

Participants who were OwOb were older, were more frequently alcohol users (N = 10,047), and had higher systolic BP, diastolic BP, LDL-C, waist circumference, and a worse metabolic profile than people with a normal weight (all *p* < 0.001). Sex and smoking distributions did not differ between the two groups. Participants who were metabolically unhealthy were older, heavier, were more frequently male, and alcohol users and, as expected, had higher BP, waist circumference, and worse metabolic data than their metabolically healthy counterparts (all *p* < 0.001). Smoking was also more prevalent among the individuals who were metabolically unhealthy (*p* = 0.037).

Participants <70 years were stratified into four groups according to BMI and metabolic status: (1) normal weight/healthy metabolic status (N = 983, 8.5%), (2) normal weight/unhealthy metabolic status (N = 3604, 31.1%), (3) OwOb/healthy metabolic status (N = 438, 3.8%), and (4) OwOb/unhealthy metabolic status (N = 6560, 56.6%). Their clinical characteristics are reported in Table 1.

High LDL-C was found in 54.4% of the participants. When LDL-C was taken into account to identify people with healthy or unhealthy metabolic status, the rates were 4.5%, 34.4%, 1.7%, and 59.5%, respectively, in the four groups.

High waist circumference was much more frequent in the individuals who were OwOb than in those with a normal weight (Table 1). Waist circumference in the four groups stratified by sex is reported in Figure S3. Metabolically healthy status (no abnormal parameter) was present in 21.4% of the participants with a normal weight and in 6.3% of those who were OwOb.

**Figure 2:**
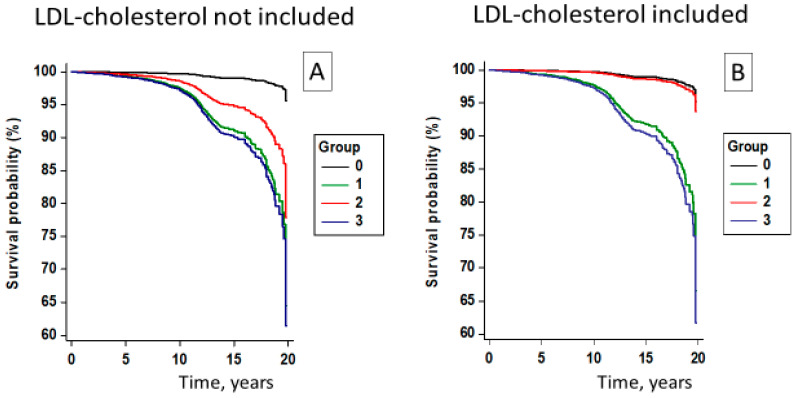
Survival curves for risk of major adverse cardiovascular events in the participants <70 years of age stratified according to BMI and metabolic status. Metabolic status was defined with standard criteria (panel (**A**)) or including LDL-cholesterol level (panel (**B**)). The risk was calculated from multivariable Cox regressions adjusted for age, sex, smoking, serum creatinine, and uric acid. Group 0 indicates participants with normal body weight and healthy metabolic status; Group 1, participants with normal body weight and unhealthy metabolic status; Group 2, participants who were overweight/obese and with healthy metabolic status; Group 3, participants who were overweight/obese and with unhealthy metabolic status.

In Figure 2 (panel A), the survival curves adjusted for age, sex, smoking, creatinine, uric acid, and LDL-C for the four groups identified using standard criteria are shown. The median (interquartile range) follow-up was 11.8 (6.0–13.5) years.

Compared to the group with normal weight/healthy metabolic status, an increased risk of MACE was found in all other groups, including the individuals classified as healthy OwOb defined according to standard criteria. The adjusted HRs were 7.56 (95% CI, 3.11–18.34, *p* < 0.001) in the metabolically unhealthy OwOb participants, 3.81 (95% CI, 1.34–10.85, *p* = 0.012) in the metabolically healthy OwOb individuals, and 6.84 (95% CI, 2.81–16.68, *p* < 0.001) in those metabolically unhealthy with a normal weight (Table S3). Further adjustments for antihypertensive treatment and BP category did not change these relationships; in the metabolically healthy OwOb individuals, the adjusted HR was 3.87 (95% CI, 1.36–11.02, *p* = 0.011). Exclusion of subjects with diabetes from the two metabolically unhealthy groups provided similar results (Table S4). Male sex, age, smoking, serum creatinine, and uric acid were all strong predictors of MACE (all *p* < 0.001) irrespective of whether LDL-C was included or not as a covariate in the regression. LDL-C showed a moderate association with MACE (*p* = 0.018) and caused a small improvement in the survival model (delta AIC, 3.58) with virtually no change in delta BIC (−0.97).

### 3.2. Models Including Variables with Missing Data

In the participants in whom information on alcohol use and usual physical activity was available, data were further adjusted for alcohol (N = 10,047), for physical activity (N = 8124), and for both (N = 7110). The results did not substantially differ from those obtained in the whole population (Tables S5–S7). Similar results were obtained also when waist circumference was accounted for (Table S8). In this model, high waist circumference was not an independent predictor of MACE, irrespective of whether LDL-C was included (HR, 1.25 (95% CI, 0.97–1.61, *p* = 0.080) or not (HR, 1.15 (95% CI, 0.91–1.45, *p* = 0.25) in the model.

When the CV risk was assessed separately in the overweight subjects and in the subjects with obesity, the association with outcome was significant for both metabolically healthy groups either when subjects with diabetes were included in the regression (Table 2) or were excluded from the model (Table S9).

The CV risk according to body weight/metabolic status showed a similar trend in men and women.

### 3.3. Metabolically Healthy Status including Normal LDL-Cholesterol Level

Participants with normal LDL-C (<130 mg/dL) were 1.9% of the total population and 3.1% of the OwOb individuals. Compared to the subjects with higher LDL-C, they were younger (*p* < 0.001) and had lower diastolic BP (*p* = 0.002) and triglycerides (*p* < 0.001), and higher HDL-cholesterol (*p* < 0.001). BMI, waist circumference, and glucose did not differ according to LDL-C level. When normal LDL-C was included in the definition of a healthy metabolic status, the metabolically healthy OwOb group had a risk of MACE similar to that of the metabolically healthy normal-weight participants (Figure 2B and Table 3). Further adjustments for antihypertensive treatment and BP category did not materially change these relationships; in the metabolically healthy OwOb individuals, the HR was 0.70 (95% CI, 0.07–6.81, *p* = 0.76). Interaction analysis to evaluate the potential modifying effects of hypertension on the relationship between LDL-C exposure and CV events showed no interactive effect of hypertension diagnosis (*p* = 0.24) or BP level (*p* = 0.15) with LDL-C category on CV outcomes. These associations did not fundamentally change when subjects with diabetes were excluded from the analysis or when waist circumference was incorporated in the model.

## 4. Discussion

The results of this analysis confirm that the relationship of BMI and metabolic status with MACE differs according to age. In people younger than 70 years, high BMI and unhealthy metabolic status were associated with an increased risk of CV disease, whereas both variables were not predictors of adverse outcomes in older people. In the younger group, OwOb status was an independent predictor of MACE, also in the absence of metabolic abnormalities, though the risk was lower than in their metabolically unhealthy counterparts. However, when normal LDL-C was included in the definition of healthy metabolic status, OwOb status was no longer associated with an increase in risk.

### 4.1. Prevalence of MHO

Due to a lack of standardized criteria, the prevalence of MHO has shown wide variation across different studies [1,2,3,4,5,6]. Major sources of variability were inconsistency in the definition of MHO and a lack of uniform cut-off values used to identify abnormal metabolic parameters. According to a meta-analysis by Rey-López et al. [23], the prevalence of MHO ranged between 6% and 75%. Applying similar criteria to those used in the present report to define MHO, two large studies performed in Europe [24] and the United States [25] reported a prevalence of MHO ranging from 12% to 17%. In a meta-analysis by Huang et al. [26], an even lower prevalence of MHO was reported in the general population (7.27%). The 6.3% frequency of individuals with healthy OwOb status found in the present study is at the lowest extreme of the range reported in the literature. This low prevalence is probably due to the strict criteria we used to identify individuals with metabolically healthy status and to the characteristics of the URRAH population, which consisted mainly of subjects with hypertension [17]. Indeed, among the participants with unhealthy metabolic status, high BP was present in 67.3% of the subjects, whereas the presence of abnormal metabolic parameters ranged from 21.7% to 28.6%. It should be pointed out that the present results mainly apply to European populations as the prevalence of obesity in our sample was 18.4%, similar to that found in other Italian (18%) [27] or European populations (16.7% to 18.6%) [24,28], which are much lower than in U.S. populations (28%) [29].

### 4.2. Association of MHO with CV Risk

Whether MHO is associated with an increased CV risk has been the subject of much debate due to the different criteria used to identify this condition across the published studies. In a meta-analysis of 22 prospective studies, people with MHO, defined by the absence of metabolic syndrome, were at an increased risk of MACE compared with participants with a normal weight and without metabolic syndrome [1]. In a more recent meta-analysis of 19 Asian cohorts, Huang et al. showed that participants with MHO had a significantly higher risk of CV disease and a borderline significantly higher risk of all-cause mortality than individuals who were metabolically healthy with a normal weight [26]. Also, the studies included in this meta-analysis used broad criteria to define MHO. Only a few studies explored the association of MHO, defined with strict criteria, with CV risk. In three out of the 22 studies considered by the Ecker et al. meta-analysis, MHO was defined as the absence of all metabolic abnormalities [1]. A pooled analysis of these three studies also showed that in the participants with obesity without any metabolic abnormality, the CV risk was increased in comparison with the normal-weight reference group. In a study by Caleyachetty et al., obtained in a cohort of 3.5 million individuals 18 years of age or older [30], individuals who were either overweight or obese with no metabolic abnormalities had a 49% increased risk of coronary heart disease, a 7% increased risk of cerebrovascular disease, and a 96% increased risk of heart failure compared to individuals with a normal weight and no metabolic abnormalities [30]. The present results obtained from a population of hypertensive and high-risk patients are consistent with those data showing that metabolically healthy OwOb individuals had an increased risk of MACE even in the absence of metabolic abnormalities. This association held true when data were adjusted for waist circumference, alcohol consumption, and physical activity habits irrespective of whether patients with diabetes were included or not in the survival models. However, participants with metabolic abnormalities had a much higher risk of adverse outcomes than the metabolically healthy groups even if their BMI was normal.

### 4.3. The Role of LDL-Cholesterol in the Association between OwOb Status and MACE

The association of LDL-C with MACE has been known for a long time [31]. A direct action of the cholesterol particles on the arterial wall which promotes the development of atherosclerotic plaques has been documented in several studies [32]. A direct relationship between LDL-C and MACE was confirmed by the present results. When LDL-C was included as a covariate in the Cox regression, there was a modest improvement in the survival model with a delta AIC of 3.58 and no improvement of BIC, and MHO defined according to standard parameters remained an independent predictor of adverse CV outcomes. However, when metabolic status in the OwOb participants was defined including LDL-C level, no increase in risk was found in the metabolically healthy OwOb participants with low LDL-C levels compared to the healthy subjects with a normal weight, suggesting that to identify a truly healthy metabolic status in an obese subject LDL-C should also be considered.

Although prior research has shown that LDL-C is a predictor of MACE, their association appears to be complicated and is likely to be affected by other factors. Indeed, data from the literature provide evidence for a role of LDL-C as a factor that may modulate the interplay between obesity, hypertension, metabolic status, and CV disease. In a Japanese study, LDL-C was a predictor for the development of metabolic syndrome with a HR of 1.24 (95% CI, 1.10–1.40; *p* < 0.001) for each one SD increase in LDL-C level [14]. In the Brisighella Heart study, suboptimal LDL-C levels, in tandem with elevated serum uric acid, were associated with an increased risk of developing metabolic syndrome [33]. Several studies have shown that about 50% of subjects with MHO may develop one or more abnormal metabolic parameters over time, transitioning to metabolically unhealthy obesity [34,35,36]. A recent analysis of the Framingham Offspring study has shown that over two thirds of participants with MHO at baseline (mean age, 57.3 years) transitioned to an unhealthy metabolic status over a 12.9-year observational period [8].

LDL-C may be a driving factor in the progression from MHO to metabolically unhealthy obesity as shown by Li et al. in a population-based cohort study [37]. In that study, the incidence of CV events in obese people who transitioned to metabolically unhealthy obesity was significantly higher than that of subjects with a normal weight and no metabolic abnormalities and similar to that of individuals with stable metabolically unhealthy obesity.

Other factors can account for the association between LDL-C and CV risk. LDL-C is thought to function as a molecular switch to initiate inflammation [38]. High LDL-C promotes cholesterol accumulation in macrophages and other immune cells, leading to inflammatory responses in the artery wall [39]. Previous research has shown that in high-risk patients, high-sensitivity C-reactive protein was a stronger predictor of CV risk than LDL-C, reinforcing the clinical concept that LDL-C and inflammation concur to determine CV events. Thus, lower levels of inflammation may be the linchpin for healthy metabolic status, including low LDL-C, with favourable metabolic consequences for OwOb subjects. In a study in which LDL-C was included in the definition of MHO, participants with MHO had comparable levels of inflammatory markers hsCRP and sICAM-1 to those of individuals with a normal weight/metabolically healthy status [13]. Given that the inflammatory response is thought to be important for the development of hypertension [40,41], we hypothesized that hypertension was an important player in the relationship between LDL-C and CV disease. However, in the present study, the interactive effect of LDL-C and hypertension on MACE did not achieve the level of statistical significance (*p* = 0.15).

Overall, our data suggest that in obesity research LDL-C should not only be included as a covariate in survival analyses but should rather be used for a more precise identification of subjects with MHO.

### 4.4. Limitations

This study has several limitations. The URRAH project is not a population-based study, but rather a cohort study of data collected on a regional basis in Italy in patients with hypertension or with high cardiovascular risk referred to tertiary centres because of the complexity of their clinical situation. Thus, the results cannot be directly applied to populations with different characteristics and to other ethnicities. This is a retrospective analysis, and BMI and metabolic variables were measured only at baseline. Therefore, we could not estimate the influence of follow-up changes in BMI and metabolic status on MACE. Information on alcohol use, physical activity, and waist circumference was not available in all subjects, and thus the influence of these factors on the combined CV effect of OwOb and metabolic status could not be tested in the whole sample. However, adjustments for these variables provided similar data to those in the whole population. Finally, markers of inflammation were not available to prove our hypothesis that inflammation plays an important role in the association between LDL-C and CV disease.

## 5. Conclusions

In keeping with the majority of the previous studies, the present findings showed that metabolically healthy overweight/obesity evaluated using standard criteria was associated with an increased risk of MACE even when baseline BP, glucose, HDL-cholesterol, and triglycerides were all normal. However, the CV risk was smaller than in subjects with metabolically unhealthy OwOb and even lower than in individuals with a normal weight and metabolically unhealthy status. LDL-C appeared as a crucial factor, influencing the diagnosis of MHO, as it allowed researchers to identify a subgroup of OwOb individuals with no increase in CV risk. This finding indicates that, in people with obesity younger than 70 years, a more comprehensive definition of MHO should be used, including LDL-C. A low inflammation state has been shown in people with a healthy metabolic state when including low LDL-C in the definition [13], and this may partly explain the reduced risk of adverse CV outcomes in these individuals. Future studies are needed to better understand the nature of these associations and the implications this could have for the management of OwOb individuals.

## Figures and Tables

**Table 1:** Characteristics of study participants <70 years of age grouped according to body mass index and metabolic status.

	BMI < 25 kg/m^2^				BMI ≥ 25 kg/m^2^				
	Metab− N = 983		Metab+ N = 3604		Metab− N = 438		Metab+ N = 6560		
Variable	Mean	SD	Mean	SD	Mean	SD	Mean	SD	*p*-Value
Age, years	40.9	11.7	51.5	12.1	46.9	12.1	54.1	10.9	<0.001
BMI, kg/m^2^	21.7	2.0	22.7	1.7	27.9	3.0	29.1	3.5	<0.001
Office SBP, mmHg	114.6	8.8	139.2	21.7	117.1	8.2	141.8	21.0	<0.001
Office DBP, mmHg	71.9	6.7	84.7	12.6	74.1	6.2	86.0	12.3	<0.001
Heart rate, bpm	68.6	10.8	72.4	12.5	67.7	10.4	72.4	12.2	<0.001
Glucose, mg/dL	82.5	7.2	92.1	16.4	85.2	7.4	98.2	22.2	<0.001
Triglycerides, mg/dL	73.9	27.3	106.8	60.6	83.9	30.4	135.9	79.3	<0.001
Uric acid, mg/dL	4.2	1.0	4.5	1.3	4.7	1.3	5.1	1.3	<0.001
Total cholesterol, mg/dL	200.8	37.7	209.4	39.6	208.4	37.5	215.9	38.8	<0.001
HDL-cholesterol, mg/dL	63.4	13.4	55.6	15.1	59.1	12.4	50.1	13.1	<0.001
LDL-cholesterol, mg/dL	122.7	34.9	132.4	35.5	132.4	34.5	138.5	35.8	<0.001
Sex, males, %	42.2	----	49.3	----	47.0	----	49.0	----	0.001
Smoker or ex-smoker, %	33.9	----	36.8	----	34.9	----	37.3	----	0.17
Anti-HT treatment, %	8.8	----	28.1	----	17.6	----	37.8	----	<0.001
Alcohol, yes (N = 10,047), %	35.6	----	53.9	----	44.7	----	59.2	----	<0.001
Sedentary, (N = 8124), %	50.9	----	58.6	----	50.3	----	61.6	----	<0.001
High waist circ, (N = 8113), %	2.4	----	7.5	----	32.2	----	44.0	----	<0.001
MACE, %	0.5	----	5.7	----	2.7	----	7.4	----	<0.001

BMI indicates body mass index; Metab−, subjects with no abnormal metabolic parameter; Metab+, subjects with at least one abnormal metabolic parameter; SBP, systolic blood pressure; DBP, diastolic blood pressure; Anti-HT, antihypertensive; MACE, major adverse cardiovascular events; circ, circumference. *p*-values for continuous variables from ANCOVA, adjusted for age and sex.

**Table 2:** Multivariable Cox model for risk of major adverse cardiovascular events in the participants <70 years of age categorized according to BMI (3 categories) and metabolic status.

Group	Estimate	Standard Error	Wald Chi^2^	*p*-Value	Hazard Ratio	95% Confidence Limit
BMI < 25.0 Metab+	1.93	0.45	18.12	<0.0001	6.92	2.84 to 16.86
BMI = 25.0–29.9 Metab−	1.22	0.56	4.75	0.029	3.38	1.13 to 10.11
BMI = 25.0–29.9 Metab+	1.95	0.45	18.57	<0.0001	7.06	2.90 to 17.18
BMI ≥ 30 Metab−	1.85	0.73	6.38	0.011	6.34	1.51 to 26.56
BMI ≥ 30 Metab+	2.20	0.46	23.13	<0.0001	9.00	3.67 to 22.04

The normal weight/metabolically healthy group was used as a reference. Data are adjusted for age, sex, smoking, LDL-cholesterol, serum creatinine, and uric acid. BMI indicates body mass index in kg/m^2^; Metab+, metabolically unhealthy; Metab−, metabolically healthy.

**Table 3:** Multivariable Cox model for risk of major adverse cardiovascular events in the participants <70 years of age categorized according to BMI (2 categories) and metabolic status defined according to standard criteria (without including LDL-cholesterol) or including LDL-cholesterol.

Group	Wald Chi^2^	*p*-Value	Hazard Ratio	95% Confidence Limit
BMI < 25 and metabolically unhealthy				
Without including LDL-CT	18.14	<0.0001	6.91	2.40 to 16.83
Including LDL-CT	7.17	0.007	4.77	1.52 to14.95
BMI ≥ 25 and metabolically healthy				
Without including LDL-CT	6.68	0.011	3.85	1.35 to 10.96
Including LDL-CT	0.07	0.79	0.73	0.08 to 7.04
BMI ≥ 25 and metabolically unhealthy				
Without including LDL-CT	19.01	<0.0001	7.19	2.96 to 17.45
Including LDL-CT	8.11	0.004	5.25	1.58 to 16.44

The normal weight/metabolically healthy group was used as a reference. Data are adjusted for age, sex, smoking, serum creatinine, and uric acid. BMI indicates body mass index in kg/m^2^; LDL-CT, LDL-cholesterol.

## Data Availability

The data that support the findings of this study are available on reasonable request from the URRAH investigators.

## Supplementary Materials

The following supporting information can be downloaded at: https://www.mdpi.com/article/10.3390/diagnostics14131314/s1, Table S1. Characteristics of the URRAH participants grouped according to age (N=15,904). Table S2. Multivariable Cox model for risk of major adverse cardiovascular events in the participants stratified by age and categorized according to BMI (3 categories). Table S3. Multivariable Cox model for risk of major adverse cardiovascular events in the participants <70 years of age categorized according to BMI (2 categories) and metabolic status including diabetic subjects. Table S4. Multivariable Cox model for risk of major adverse cardiovascular events in the participants <70 years of age categorized according to BMI (2 categories) and metabolic status excluding diabetic subjects. Table S5. Multivariable Cox model for risk of major adverse cardiovascular events in the participants <70 years of age categorized according to BMI (2 categories) and metabolic status including diabetic subjects. Table S6. Multivariable Cox model for risk of major adverse cardiovascular events in the participants <70 years of age categorized according to BMI (2 categories) and metabolic status including diabetic subjects. Table S7. Multivariable Cox model for risk of major adverse cardiovascular events in the participants <70 years of age categorized according to BMI (2 categories) and metabolic status including diabetic subjects. Table S8. Multivariable Cox model for risk of major adverse cardiovascular events in the participants <70 years of age categorized according to BMI (2 categories) and metabolic status. Table S9. Multivariable Cox model for risk of major adverse cardiovascular events in the participants <70 years of age categorized according to BMI (3 categories) and metabolic status excluding diabetic subjects. Figure S1. Distribution of body mass index in 15,904 URRAH participants stratified by age. Figure S2. Distribution of body mass index in the URRAH participants <70 years of age stratified by metabolic status. Figure S3. Waist circumference in the participants <70 years of age grouped according to body mass index (BMI) and metabolic status.
