# Supplementary material for: Risk of Cardiovascular Events in Metabolically Healthy Overweight or Obese Adults: Role of LDL-Cholesterol in the Stratification of Risk
Source: Diagnostics (Basel). 2024 Jun 21;14(13):1314. doi: 10.3390/diagnostics14131314 (PMC11240609; doi:10.3390/diagnostics14131314)
Supplement: Supplementary file 1 [file diagnostics-14-01314-s001.zip › diagnostics-3005499-supplementary.pdf]

Supplementary tables and figures

RISK OF CARDIOVASCULAR EVENTS IN ADULTS WITH METABOLICALLY HEALTHY OVERWEIGHT OR OBESITY: ROLE OF LDL-CHOLESTEROL IN THE STRATIFICATION OF RISK.

Authors: Paolo Palatini, Agostino Virdis, Stefano Masi, Alessandro Mengozzi, Edoardo Casiglia, Valerie Tikhonoff, Arrigo F.G. Cicero, Andrea Ungar, Gianfranco Parati, Giulia Rivasi, Massimo Salvetti, Carlo M. Barbagallo, Michele Bombelli, Raffaella Dell'Oro, Berardino Bruno, Luciano Lippa, Lanfranco D'Elia, Maria Masulli, Paolo Verdecchia, Gianpaolo Reboldi, Fabio Angeli, Rosario Cianci, Francesca Mallamaci, Massimo Cirillo, Marcello Rattazzi, Pietro Cirillo, Loreto Gesualdo, Elisa Russo, Alberto Mazza, Cristina Giannattasio, Alessandro Maloberti, Massimo Volpe, Giuliano Tocci, Guido Iaccarino, Pietro Nazzaro, Ferruccio Galletti, Claudio Ferri, Giovambattista Desideri, Francesca Viazzi, Roberto Pontremoli, Maria Lorenza Muiesan, Guido Grassi, Claudio Borghi from the Working Group on Uric Acid and Cardiovascular Risk of the Italian Society of Hypertension (SIIA).

Table S1. Characteristics of the URRAH participants grouped according to age (N=15,904).

Variable	Age < 70 years N=11585		Age ≥ 70 years N=4319		
	Mean	SD	Mean	SD	p-value*
Age, years	51.9	12.0	75.7	4.8	< 0.001
Body mass index, Kg/m^2	26.5	4.3	26.7	4.3	0.002
Office systolic BP, mmHg	137.8	21.9	155.1	24.4	< 0.001
Office diastolic BP, mmHg	83.9	12.6	84.4	12.4	0.049
Heart rate, bpm	71.9	12.2	72.0	11.8	0.61
Glucose, mg/dL	94.0	19.6	105.0	29.9	< 0.001
Triglycerides, mg/dL	119.6	72.3	117.5	53.6	0.11
Uric acid, mg/dL	4.8	1.3	5.2	1.4	< 0.001
Total cholesterol, mg/dL	212.3	39.2	212.1	39.8	0.81
HDL-cholesterol, mg/dL	53.3	14.3	56.1	15.5	< 0.001
LDL-cholesterol, mg/dL	135.0	35.9	132.5	35.7	< 0.001
Waist circumference, cm†	88.7	12.6	94.3	12.0	< 0.001
Sex, males	48.5%	----	46.4%	----	0.02
Hypertension, yes	60.0%	----	83.0%	----	<0.001
HT stage 1	60.3%	----	54.9%	----	
HT stage 2	39.7%	----	45.1%	----	
AntiHT therapy	31.6%	----	31.4%	----	0.85
Diabetes, yes	7.3%	----	16.6%	----	<0.001

MACE	6.1%	----	24.2%	----	<0.001

*Adjusted for sex. †N = 10,112; BP indicates blood pressure; AntiHT therapy, antihypertensive therapy; MACE, major adverse cardiovascular events; HT stage 1, stage 1 hypertension; HT stage 2, stage 2 hypertension (according to the 2023 ESH guidelines).

Table S2. Multivariable Cox model for risk of major adverse cardiovascular events in the participants stratified by age and categorized according to BMI (3 categories). The normal weight group was used as a reference.

BMI Group	Age group	Wald Chi^2	p-value	Hazard Ratio	95% Confidence limit
**BMI≥25 <30 kg/m2**	< 70 years	1.25	0.26	1.10	0.93 to 1.29
	≥ 70 years	12.22	<0.001	0.80	0.70 t0 0.91
**BMI≥30 Kg/m2**	< 70 years	10.26	0.001	1.37	1.29 to 1.66
	≥ 70 years	3.67	0.055	0.85	0.72 to 1.00

Data are adjusted for age, sex, smoking, metabolic status, total cholesterol, serum creatinine and uric acid. BMI indicates body mass index in Kg/m^2.

Table S3. Multivariable Cox model for risk of major adverse cardiovascular events in the participants < 70 years of age categorized according to BMI (2 categories) and metabolic status including diabetic subjects.

Group	Estimate	Standard error	Wald Chi^2	p-value	Hazard Ratio	95% Confidence limit
**BMI<25.0 Metab +**	1.92	0.45	17.92	<0.0001	6.84	2.81 to 16.68
**BMI≥25 Metab -**	1.34	0.53	6.30	0.012	3.81	1.34 to 10.85
**BMI≥25 Metab +**	2.02	0.45	20.00	<0.0001	7.56	3.11 to 18.35

The normal weight/metabolically healthy group was used as a reference. Data are adjusted for age, sex, smoking, total cholesterol, serum creatinine and uric acid. BMI indicates body mass index in Kg/m^2; Metab +, metabolically unhealthy; Metab - , metabolically healthy.

Table S4. Multivariable Cox model for risk of major adverse cardiovascular events in the participants < 70 years of age categorized according to BMI (2 categories) and metabolic status excluding diabetic subjects.

Group	Estimate	Standard error	Wald Chi^2	p-value	Hazard Ratio	95% Confidence limit
**BMI<25.0 Metab +**	1.92	0.45	17.92	<0.0001	6.84	2.81 to 16.68
**BMI≥25 Metab -**	1.34	0.53	6.30	0.012	3.81	1.34 to 10.84
**BMI≥25 Metab +**	2.02	0.46	19.99	<0.0001	7.56	3.11 to 18.35

The normal weight/metabolically healthy group was used as a reference. Data are adjusted for age, sex, smoking, total cholesterol, serum creatinine and uric acid. BMI indicates body mass index in Kg/m^2; Metab +, metabolically unhealthy; Metab - , metabolically healthy.

Table S5. Multivariable Cox model for risk of major adverse cardiovascular events in the participants < 70 years of age categorized according to BMI (2 categories) and metabolic status including diabetic subjects. Data were adjusted also for alcohol use. N=10,047

Group	Estimate	Standard error	Wald Chi^2	p-value	Hazard Ratio	95% Confidence limit
**BMI<25.0 Metab +**	2.65	0.71	13.87	0.0002	14.20	3.51 to 57.35
**BMI≥25 Metab -**	2.01	0.79	6.43	0.011	7.44	1.58 to 35.07
**BMI≥25 Metab +**	2.73	0.71	14.77	0.0001	15.36	3.81 to 61.87

The normal weight/metabolically healthy group was used as a reference. Data are adjusted for age, sex, smoking, total cholesterol, serum creatinine, uric acid and alcohol use. BMI indicates body mass index in Kg/m^2; Metab +, metabolically unhealthy; Metab - , metabolically healthy.

Table S6. Multivariable Cox model for risk of major adverse cardiovascular events in the participants < 70 years of age categorized according to BMI (2 categories) and metabolic status including diabetic subjects. Data were adjusted also for physical activity. N=8,124.

Group	Estimate	Standard error	Wald Chi^2	p-value	Hazard Ratio	95% Confidence limit
**BMI<25.0 Metab +**	1.805	0.59	9.45	0.002	6.08	1.92 to 19.24
**BMI≥25 Metab -**	1.520	0.68	5.02	0.025	4.57	1.21 to 17.26
**BMI≥25 Metab +**	1.89	0.58	10.48	0.001	6.63	2.11 to 20.85

The normal weight/metabolically healthy group was used as a reference. Data are adjusted for age, sex, smoking, total cholesterol, serum creatinine, uric acid and usual physical activity. BMI indicates body mass index in Kg/m^2; Metab +, metabolically unhealthy; Metab - , metabolically healthy.

Table S7. Multivariable Cox model for risk of major adverse cardiovascular events in the participants < 70 years of age categorized according to BMI (2 categories) and metabolic status including diabetic subjects. Data were adjusted also for alcohol use and physical activity. N=7,110.

Group	Estimate	Standard error	Wald Chi^2	p-value	Hazard Ratio	95% Confidence limit
**BMI<25.0 Metab +**	2.04	0.72	8.12	0.004	7.70	1.89 to 31.34
**BMI≥25 Metab -**	1.77	0.82	4.67	0.031	5.85	1.18 to 29.05
**BMI≥25 Metab +**	2.14	0.71	8.97	0.003	8.48	2.09 to 34.36

The normal weight/metabolically healthy group was used as a reference. Data are adjusted for age, sex, smoking, total cholesterol, serum creatinine, uric acid, alcohol use and usual physical activity. BMI indicates body mass index in Kg/m^2; Metab +, metabolically unhealthy; Metab - , metabolically healthy.

Table S8. Multivariable Cox model for risk of major adverse cardiovascular events in the participants < 70 years of age categorized according to BMI (2 categories) and metabolic status. Data were adjusted also for waist circumference. N=8,113

Group	Estimate	Standard error	Wald Chi^2	p-value	Hazard Ratio	95% Confidence limit
**BMI<25.0 Metab +**	2.04	0.72	8.12	<0.001	8.52	2.70 to 27.00
**BMI≥25 Metab -**	1.77	0.82	4.67	0.030	4.35	1.15 to 16.46
**BMI≥25 Metab +**	2.14	0.71	8.97	<0.001	7.15	2.27 to 22.57

The normal weight/metabolically healthy group was used as a reference. Data are adjusted for age, sex, smoking, total cholesterol, serum creatinine, uric acid, and waist circumference. BMI indicates body mass index in Kg/m^2; Metab +, metabolically unhealthy; Metab - , metabolically healthy.

Table S9. Multivariable Cox model for risk of major adverse cardiovascular events in the participants < 70 years of age categorized according to BMI (3 categories) and metabolic status excluding diabetic subjects.

Group	Estimate	Standard error	Wald Chi^2	p-value	Hazard Ratio	95% Confidence limit
**BMI<25.0 Metab +**	1.89	0.45	17.21	<0.0001	6.60	2.70 to 16.10
**BMI=25.0-29.9 Metab -**	1.19	0.56	4.52	0.034	3.28	1.10 to 9.82
**BMI=25.0-29.9 Metab +**	1.82	0.45	16.0	0.0001	6.16	2.53 to 15.02
**BMI≥30 Metab -**	1.84	0.73	6.31	0.012	6.28	1.50 to 26.31
**BMI≥30 Metab +**	1.97	0.46	18.45	<0.0001	7.20	2.92 to 17.72

The normal weight/metabolically healthy group was used as a reference. Data are adjusted for age, sex, smoking, total cholesterol, serum creatinine and uric acid. BMI indicates body mass index in Kg/m^2; Metab +, metabolically unhealthy; Metab - , metabolically healthy

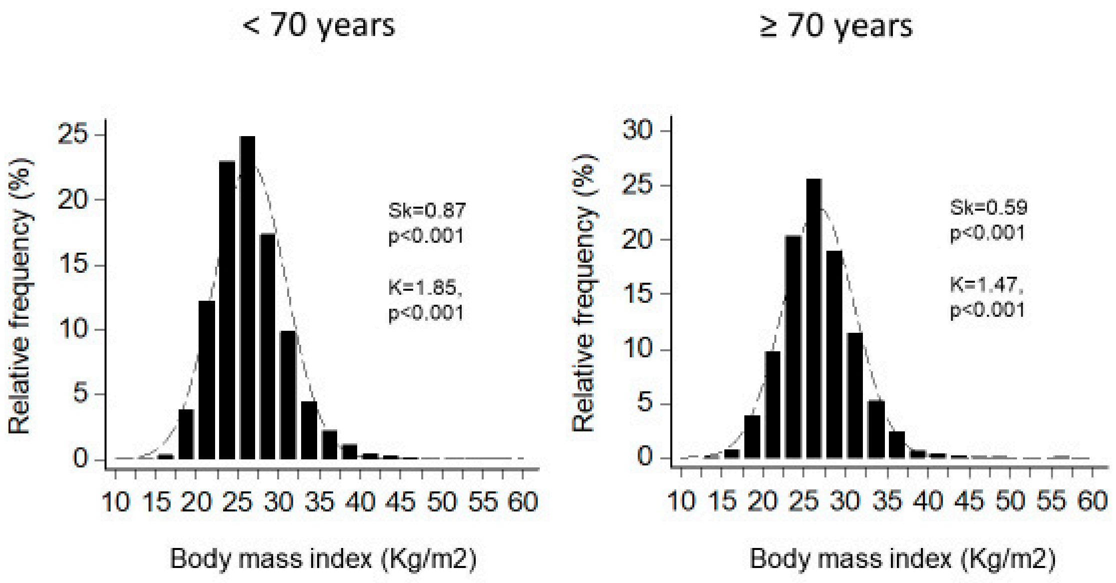

Figure S1. Distribution of body mass index in 15,904 URRAH participants stratified by age. Sk indicates coefficient of skewness; K, coefficient of Kurtosis.

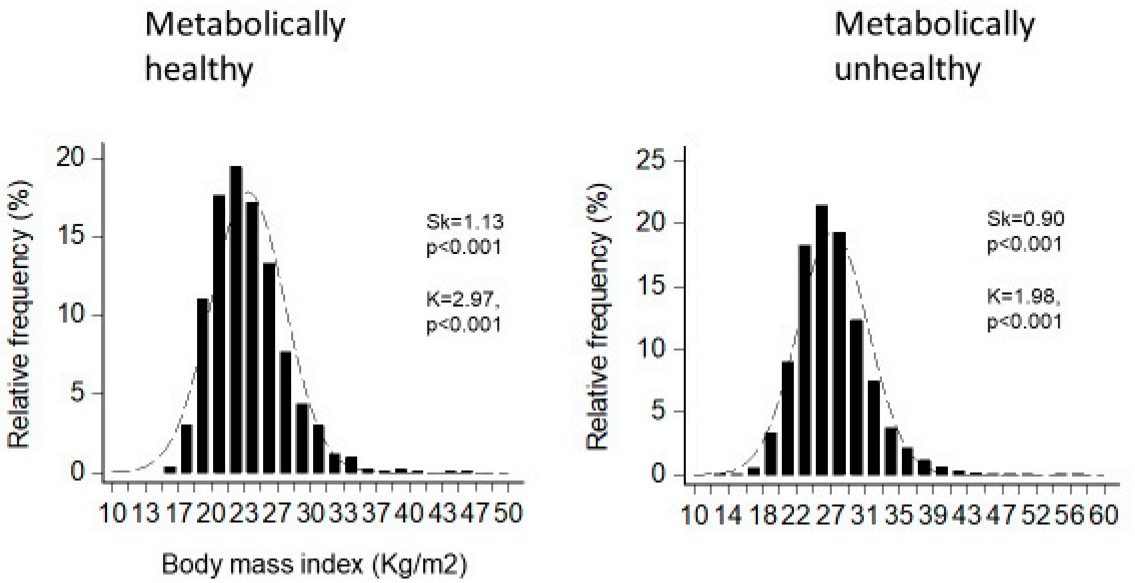

Figure S2. Distribution of body mass index in the URRAH participants < 70 years of age stratified by metabolic status. Sk indicates coefficient of skewness; K, coefficient of Kurtosis.

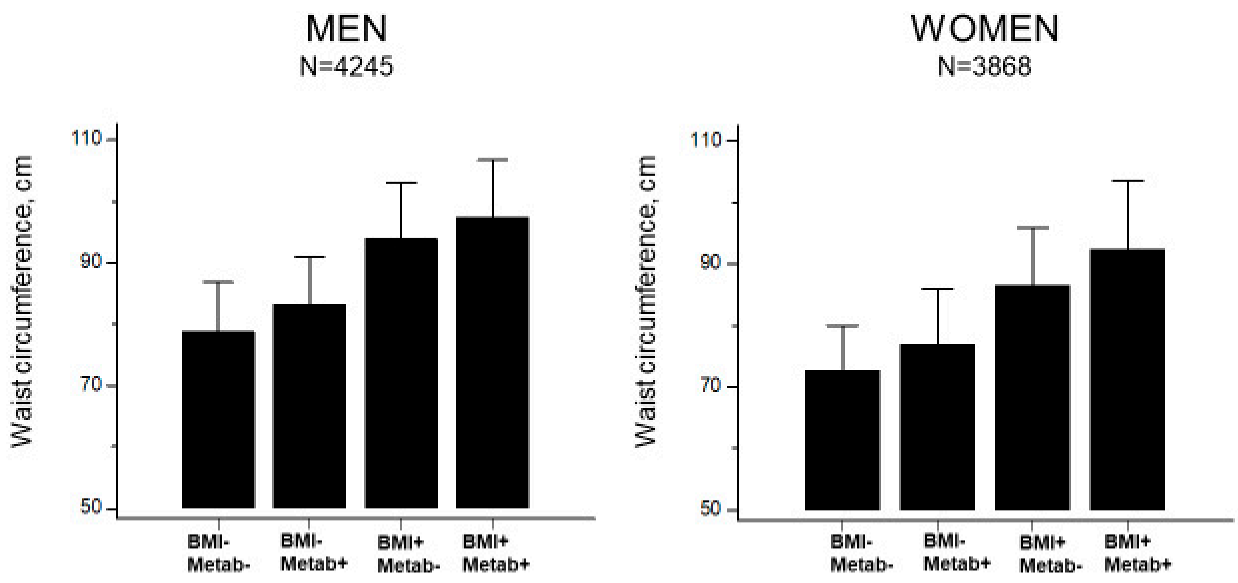

Figure S3. Waist circumference in the participants < 70 years of age grouped according to body mass index (BMI) and metabolic status. BMI- indicates participants with BMI<25 kg/m^2; BMI+ indicates participants with BMI≥ 25Kg/m^2 ; Metab- indicates participants with healthy metabolic status; Metab+, indicates participants with unhealthy metabolic status. Age-adjusted P from two-way ANCOVA: P for group < 0.0001; P for sex < 0.0001; P for group x sex interaction < 0.0001.
